# The Effect of Matrix Composition on the Deformation and Failure Mechanisms in Metal Matrix Syntactic Foams during Compression

**DOI:** 10.3390/ma10020196

**Published:** 2017-02-17

**Authors:** Csilla Kádár, Kristián Máthis, Michal Knapek, František Chmelík

**Affiliations:** 1 Department of Materials Physics, Loránd Eötvös University, Pázmány P. stny. 1/A, Budapest H-1117, Hungary; kadar@metal.elte.hu; 2 Department of Physics of Materials, Charles University, Ke Karlovu 5, Prague 2 121 16, Czech Republic; mathis@met.mff.cuni.cz (K.M.); chmelik@met.mff.cuni.cz (F.C.)

**Keywords:** syntactic foams, acoustic emission, mechanical properties

## Abstract

The influence of the matrix material on the deformation and failure mechanisms in metal matrix syntactic foams was investigated in this study. Samples with commercially pure Al (Al) and Al-12 wt % Si (AlSi12) eutectic aluminum matrix, reinforced by hollow ceramic spheres, were compressed at room temperature. Concurrently, the acoustic emission response and the strain field development on the surface were monitored in-situ. The results indicate that the plastic deformation of the cell walls is the governing mechanism in the early stage of straining for both types of foams. At large stresses, deformation bands form both in the Al and AlSi12 foam. In Al foam, cell walls collapse in a large volume. In contrast, the AlSi12 foam is more brittle; therefore, the fracture of precipitates and the crushing of the matrix take place within a distinctive deformation band, along with an occurrence of a significant stress drop. The onset stress of ceramic sphere failure was shown to be not influenced by the matrix material. The in-situ methods provided complementary data which further support these results.

## 1. Introduction

In the past decades, man-made materials with a relatively high volume fraction of void space (i.e., a low relative density), inspired by lightweight cellular structures found in nature, have drawn attention and many researchers have focused on their manufacture and characterization [1,2]. Porous materials can advantageously combine the properties of the material they are made of with their structural properties to produce materials for certain applications, such as energy absorption, lightweight structures, mechanical damping, and filtering [2,3]. Among porous materials, cellular metals feature a relatively high plateau stress (unlike polymer foams), high ductility (unlike porous ceramics), and durability. Among the above-mentioned applications, they can be also suitable for the protection against impact and blasts in the form of, e.g., lightweight panels, boxes, or structural parts of buildings and vehicles [4,5].

Metal matrix syntactic foams (MMS foams) are a relatively new class of materials which comprise of enclosed porosity inside stiff spheres (or “microballoons”) incorporated in the metallic matrix. Owing to these spheres, MMS foams can typically withstand higher stresses compared with conventional metal foams containing gas porosity [6]. They are, therefore, recognized particularly for their potential as energy absorbers in the transport industry or impact protection [5]. For such applications, the mechanical properties of materials play a crucial role. Many studies have addressed the mechanical behavior upon impact or loading of syntactic foams with various matrix and sphere materials (e.g., [7,8,9,10,11,12,13]). In order to effectively tailor the compressive strength and the energy absorption capacity of a given foam, the detailed understanding of internal deformation and damage mechanisms is of extremely high importance.

The failure mechanisms and damage evolution during compression of metal foams have been investigated by different methods, such as X-ray tomography [9], surface strain mapping [14] or digital macro-imaging [15]. These techniques give information on the damage on a mesoscopic scale, i.e., mainly on the cell deformation or visible fracture of the cell-walls or hollow spheres. The drawbacks of these methods are that either the time-resolution is not satisfying (as in the case of X-ray tomography) or the observation is limited to the surface of the sample.

Acoustic emission (AE) has proven to be a useful tool in investigating the failure mechanisms in numerous materials as the time-resolution of this method is in order of microseconds. Moreover, in contrast to the above-mentioned characterization methods, AE provides information continuously from the entire volume of the specimen. AE originates from transient elastic waves generated within the material as a result of sudden localized irreversible microstructural changes. Thus, it yields information on, e.g., plastic deformation and crack formation and propagation within the material and, thereby, also on the localization of the deformation.

In this paper, MMS foams with two different matrix materials, pure Al and Al-12 wt % Si, reinforced by hollow ceramic spheres of identical size distribution and volume fraction were deformed in compression at room temperature. The aim of this work was to investigate the nature of the damage during compression of these two foam types, on a micro-scale, using a recently developed AE data analysis technique Adaptive sequential k-means clustering (ASK) [16]. Using complementary techniques, i.e., microscopy, video recording, and surface strain mapping, different AE sources were successfully identified in MMS foam samples. The influence of the matrix composition on the deformation and failure mechanisms is discussed in detail. 

## 2. Materials and Methods

### 2.1. Materials

MMS foams investigated in this work were produced using the pressure infiltration technique. First, hollow ceramic spheres (produced by Hollomet GmbH, Dresden, Germany; consisting of 35 wt % Al_2_O_3_, 45 wt % SiO_2_ and 20 wt % mullite) with an average outer diameter of 1.45 mm and a wall thickness of 58 μm were poured into a steel mold and slightly compacted by swirling. Next, an alumina (Al_2_O_3_) insulator layer was placed on top of the spheres to separate the block of matrix material (placed on the insulator layer) and the spheres. After heating the mold to 710 °C, an argon pressure was applied during infiltration (30 s at 0.4 MPa). Finally, the mold with the infiltrated ceramic spheres was quenched. A more detailed description of the process can be found in [17]. By this method, approximately 64% volume fraction of spheres within the foam can be achieved [18]. Two different matrix materials, a technical purity Al 99.5 (99.5 wt % Al, 0.2 wt % Si, and 0.3 wt % Fe) (Al) and a eutectic Al-12 wt % Si alloy (AlSi12), were used in this study. The density was calculated as m/V, where m is the mass of the sample and V is the volume determined from the dimensions of the block. The density was 1.80 ± 0.01 g·cm^−3^ in both investigated materials.

### 2.2. Methods

An Instron^®^ 5882 machine (Instron, Norwood, MA, USA) was used for compressive testing. Three specimens of each type, 30 mm in length and 14 × 14 mm^2^ in cross-section, were deformed at room temperature with a constant cross-head speed of 0.03 mm·s^−1^. During compression, the stress-strain curves and the AE response were recorded. Furthermore, the surface deformation was captured by a high-resolution digital camera. Digital image correlation (DIC) technique was employed to evaluate the strain localization in the sample. *Ncorr* open-source program [19] was used for the DIC analysis.

The AE response was monitored using a computer controlled PAC PCI-2 device (Physical Acoustics Corporation, Princeton Junction, NJ, USA) operating in the waveform streaming mode (a complete, non-parameterized AE signal is stored during the recording). The AE signal from the sensor was pre-amplified with a gain of 40 dB. The transducer was mounted on the specimen by means of a clothespin and a silicon grease.

The microstructure of the foam samples was studied using an FEI QUANTA 3D dual beam scanning electron microscope (SEM) (FEI, Hillsboro, OR, USA). In order to identify cracks in different phases of the matrix, both secondary electrons and backscattered electron (BSE) images were studied. The composition of different phases was determined by Energy Dispersive X-ray (EDX) analysis.

## 3. Results and Discussion

**Figure 1:**
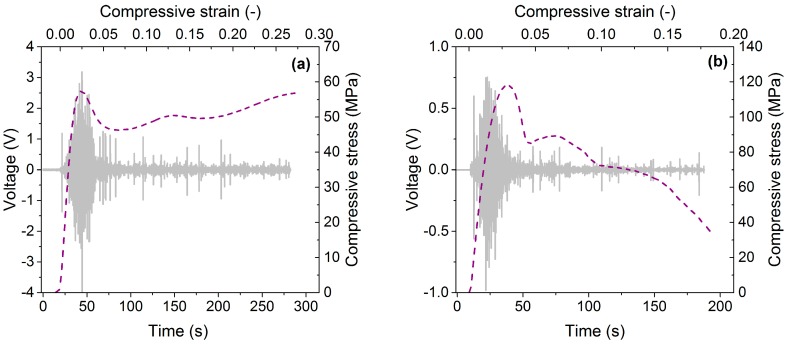
Typical compressive stress-strain curves (dashed line) and acoustic emission (AE) signals (grey line) for metal matrix syntactic (MMS) foams with (**a**) Al matrix; (**b**) AlSi12 matrix.

Typical deformation curves of Al and AlSi12 foams are plotted in Figure 1 (dashed lines). Regardless of the matrix material, the stress-strain curves show similar characteristics, i.e., after the quasi-linear region, the curves reach a peak stress, after which the stress first decreases and then behaves in a wavy manner. Conversely, the AE response depends on the matrix material. In the case of AlSi12 foam, the AE response shows maximum before the stress reaches its peak value and at the stress maximum, the AE activity is already significantly decreased (Figure 1b, continuous line). In contrast, in Al foam the AE activity is still high at peak stress. The AE response up to approximately 5% strain is higher in Al foam. It is worth noting that even in the quasi-linear region the AE activity was measured regardless of the matrix material, suggesting that “pure” elasticity does not take place it these materials (or it occurs only at very low strains). The AE behavior of the Al foam was partly investigated also in our recent work [20].

### 3.1. ASK Method

In order to reveal the failure mechanisms during compression, the AE signals were analyzed using the ASK procedure. This new clustering method was proposed by Pomponi and Vinogradov and has proven to be effective (especially when supplemented by other, mainly imaging, techniques) to correlate the AE signals with different emitting sources, based on differences in the power spectral density function (PSD) of AE signals [16]. As this method has been introduced only recently, a short description of the ASK evaluation is given in this paper. The detailed description of the algorithm can be found in [16].

The recorded AE signals are sectioned using so-called “time windows”. The PSD of the first time window is calculated and its statistical parameters (median frequency, peak value, energy, kurtosis, etc.) are determined. The first cluster (Cluster 1) is defined by these parameters. Since the AE measurement starts before the compression test, the first cluster always represents the noise. Next, PSD and the statistical parameters of the second time window are determined. If these parameters are similar to the parameters of the first element in Cluster 1, then this time window is classified as Cluster 1. If not, a new cluster (Cluster 2) is established. In the n^th^ step, PSD and its statistical parameters of the n^th^ time window are determined. If these parameters are similar to those in an already existing clusters, then the n^th^ time window is assigned to this cluster. Otherwise, a new cluster is established. In our evaluation, the size of the time window was 2 ms. We have to emphasize two important features of the ASK procedure. First, the method evaluates particular waveforms only on a statistical basis. The physical meaning of clusters must be identified by users. Second, the ASK method determines the *dominant* deformation or fracture mechanism within a “time window”. It does not exclude concurrent activity of several mechanisms; it only says which one is prevalent.

#### ASK Evaluation on Syntactic Foams

In order to identify the clusters, their characteristic features such as energy, frequency distributions and evolution of the cumulative number of elements were analyzed. Furthermore, DIC and SEM were used as complementary methods to corroborate the identification of emitting sources.

**Figure 2:**
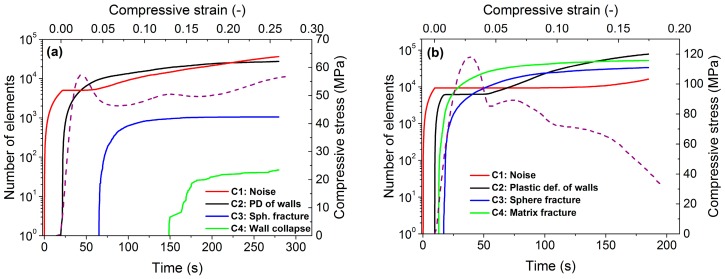
Time evolution of the cumulative number of elements in different AE clusters in (**a**) Al foam; (**b**) AlSi12 foam. Red line—noise; black—plastic deformation of matrix; blue—sphere fracture; green—Cluster 4: wall collapse in Al foam and brittle phase fracture of the matrix in AlSi12 foam. The stress-strain curve (dashed line) is also displayed.

**Figure 3:**
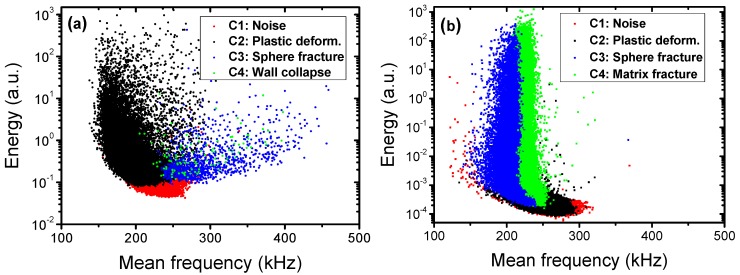
The 2D projection of the clusters to the energy-mean frequency space for (**a**) Al foam; (**b**) AlSi12 foam.

The ASK analysis algorithm identified four clusters for both Al and AlSi12 foams. Figure 2 shows the time evolution of the cumulative number of elements in the four different AE clusters. According to the time of occurrence of the cluster and its characteristic features, such as energy or mean frequency (Figure 3), the following source mechanisms were assigned to the clusters:

Cluster 1: Noise (color code in figures: red)

**Figure 4:**
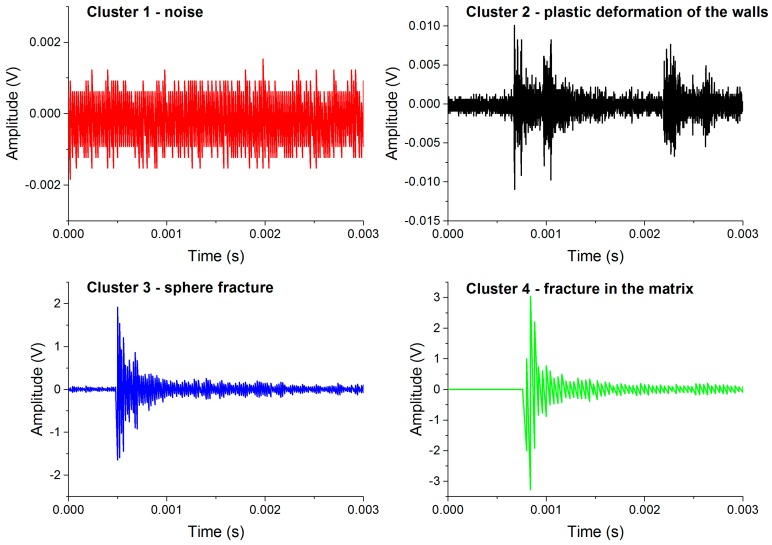
Characteristic AE waveforms corresponding to different clusters in AlSi12 foam: Cluster 1: noise; Cluster 2: plastic deformation of the walls; Cluster 3: sphere fracture; and Cluster 4: fracture in the brittle phase of the matrix.

In each test and for both materials the first cluster represents the noise. As mentioned above, the AE test starts prior to the compression test and, therefore, is unrelated to the deformation processes. Cluster 1 contains AE signals with low energy (*E* < 0.1 a.u.) (Figure 3) and “noise-shaped” signals characterized by low amplitude and no sharp peaks (Figure 4). For both samples, the number of elements in this cluster starts to increase again in the latter stages of deformation: in Al foam, this occurs at ~5% strain and in AlSi12 foam at ~10% strain (Figure 2). The effect is most likely related to the lower amount of “real” AE signals originating in the sample in the latter stages of compression, meaning that the noise component can dominate in many time windows over the scarce real signals.

Cluster 2: Plastic deformation of the metallic walls (color code in figures: black)

Cluster 2 arises at low stresses in both foams types. Its occurrence in the early stage of straining, as well as a characteristic drop shape of the cluster, indicate its origin in the plastic deformation of the metal matrix [16,20]. The evolution of this cluster is similar in both foams. However, the energy of the AE signals in AlSi12 foam is lower than in Al foam, which is a typical effect observed in alloyed materials: alloying elements and phase interfaces act as obstacles for the movement of dislocations, thus reducing their mean free path and, in turn, the amplitude and energy of the AE signals.

Cluster 3: Spheres fracture (color code in figures: blue)

**Figure 5:**
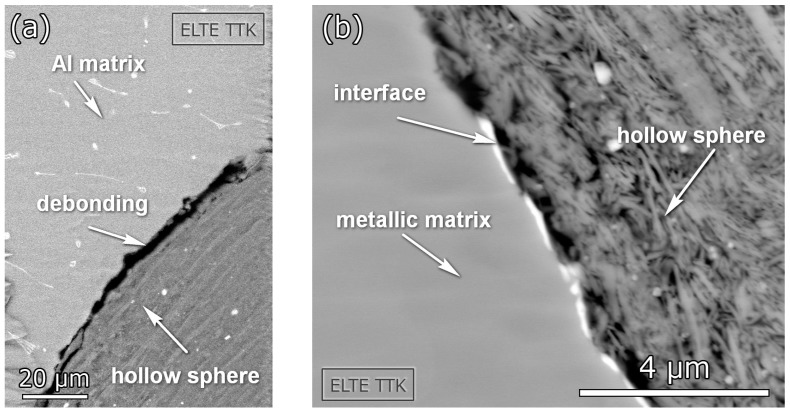
SEM micrographs showing (**a**) debonding of ceramic spheres from the Al matrix and (**b**) layered structure of the ceramic spheres.

The signals in this clusters have practically no rise time (see Figure 4), which is a characteristic signature of fracture [20,21]. Furthermore, the frequency and energy range is wide (Figure 3), suggesting that the AE source in this cluster corresponds to the fracture of ceramic spheres. During the deformation process, the load acting on the spheres varies from place to place due to deformation localization (see also DIC analysis in Section 3.2). Moreover, the hollow spheres exhibit a significant variation in their thickness [18]. This can lead to an activation of different fracture modes and irregular fracture propagation within the spheres. The debonding of spheres from the matrix is also very likely to take place at a considerable number of interfaces (see Figure 5a), which, in turn, influences the acoustic contact. All these effects lead to wide energy and frequency spectra of the signals.

Cluster 4: Collapse of the cell walls in Al-based foams; matrix fracture in AlSi12-based foams (color code in figures: green)

Cluster 4 exhibits rather different features in the two particular foam types. In the case of Al foam, this cluster appears at above 10% strain and the AE signals typically consist of a sequence of small bursts related to the collective collapse of cell walls and resulting in a formation of wide deformation bands [20]. In the case of AlSi12, however, Cluster 4 emerges even prior to the occurrence of Cluster 3. The signal waveforms are somewhat similar in these two clusters. The energy range of signal elements in Cluster 4 is wide and contains a significant high-energy component, implying that the fracture is the source of AE. On the other hand, the frequency distribution of the AE elements in this cluster is rather narrow when compared with Cluster 3. From these facts, it can be inferred that the source of AE is most likely the fracture of brittle phases in the AlSi12 matrix (the EDX analysis showed that the AlSi12 matrix contains, in addition to a pure Al phase, also Si and intermetallic Al-Si-Fe phases). The size distribution of these inclusions is relatively homogeneous compared with the thickness of ceramic layers within the walls of the hollow spheres (Figure 3b). Furthermore, the elastic wave originating in the fracture of such phases can easily propagate in the foam matrix. Thus, the scatter in the mean frequency is expected to be lower. In conclusion, the recorded AE signals in Cluster 4 result from (i) the collapse of cell walls in Al foam; (ii) the fracture of brittle phases in the AlSi12 matrix.

The results of ASK analysis show that, after a very short elastic deformation, the Al foam deforms by plastic deformation of the matrix material (while still being in the quasi-linear stage). Around the peak stress, the main deformation mechanism changes from plastic deformation to the fracture of the hollow spheres, resulting in stress drops. As can be seen on the supplementary video file [22], the falling ceramic crumbles appear also around the time when the stress reaches its maximum, confirming changes in the deformation mode (suggested also by ASK evaluation). As the deformation continues, broad deformation bands appear. These results are in line with the observations of Kiser et al. [23] who investigated A201 and A360 aluminum matrix reinforced by alumina (Al_2_O_3_) microballoons and found that the stress maxima were attained at relatively small strains, followed immediately by an onset of extensive crushing of the microballoons and associated strain localization. Deformation bands in MMS foams were observed also by other authors (e.g., [9,11,24,25]). In our work, the formation of deformation bands in Al foam is also predicted by AE as the collective collapse of ceramic spheres and cell walls results in a formation of Cluster 4 (discussed above).

**Figure 6:**
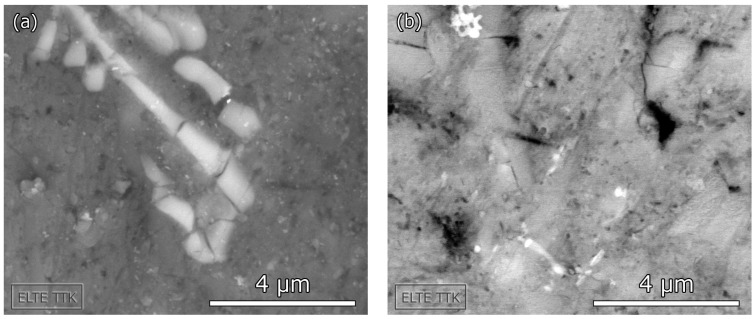
SEM images of the fractured brittle phases (**a**) Al-Si-Fe intermetallic phase; (**b**) Si phase.

The compressive strength of AlSi12 foam is higher than that of the foam with pure Al matrix and this finding agrees with previous observations [11]. The AE response at low strain is somewhat similar to that of Al foam. Plastic deformation of the matrix takes place almost as soon as the compression starts, and the AE activity increases with strain. As the stress rises, fracture-like events take place in the matrix (occurrence of Cluster 4). In order to prove this assumption, we performed SEM observations of the microstructure at this level of deformation. The SEM images in Figure 6 clearly show the fracture of brittle phases in AlSi12. Similar type of damage was observed in Al-Si eutectic alloys after cold rolling [26]. Increased strength and brittleness in MMS foams with alloyed matrix material can be explained by the strengthening effect caused by the presence of alloying elements and secondary phases formed [11].

On further compression, Cluster 3 appears which indicates that also the stiffer hollow spheres start to break. In the supplementary video file [27], falling ceramic crumbles can be seen; the first crumble can be observed shortly after the occurrence of Cluster 4 (~1% strain). Figure 2b shows that as soon as Cluster 3 appears, the main deformation mechanisms are the fracture of spheres and brittle phases, whereas the plastic deformation caused by dislocation movement becomes negligible in the AE frequency spectrum up to 5% strain. Upon further compression, the number of elements in Cluster 2, which corresponds to the plasticity of cell walls, increases again. Hence, the increasing stress after the first stress drop at 5% strain is caused by work hardening in the Al-rich phase of the matrix. The video [27] reveals that in this stage the upper and the lower parts of the foam slide on each other by means of a 45° shear band. 

The comparison of the ASK analysis results from Al and AlSi12 foam reveals that while in the case of AlSi12 foam the fracture of the hollow ceramic sphere starts at low strain (~1%), in Al foam the sphere fracture appears at higher (~5%) strain. However, a closer examination shows that in both foams the ceramic spheres start to collapse when the stress reaches ~60 MPa. In Al foam, as the spheres collapse, a high degree of plastic deformation also takes place which reduces the size of the stress drop (~10 MPa). In AlSi12 foam, there is practically no plastic deformation of the cell walls in this stage of compression, resulting in a higher stress drop of ~25 MPa.

### 3.2. DIC Analysis on Syntactic Foams

**Figure 7:**
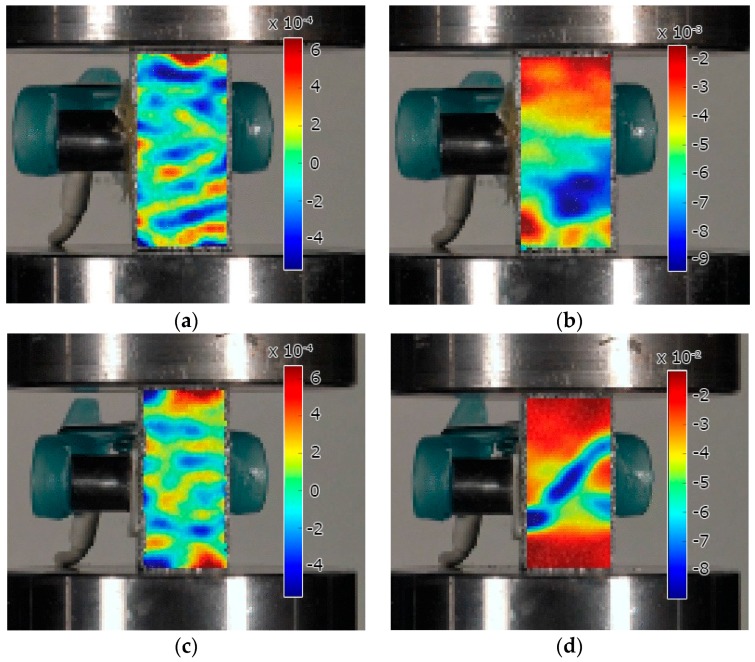
Surface *ε*_yy_ strain maps at different macroscopic strain: (**a**) Al foam at 2%; (**b**) Al foam at 4%; (**c**) AlSi12 foam at 2%; (**d**) AlSi12 foam at 4%. (The scale of *ε*_yy_ strain is different in each figure.).

The DIC technique was used in order to corroborate the results of the mechanical test and the AE analysis. The DIC images of Al foam show that the strain is only slightly localized at 2% macroscopic strain by means of randomly distributed “islands” of higher accumulated strain values (Figure 7a). Upon further compression, well-defined deformation regions are formed. Figure 7b shows that at 4% macroscopic strain the deformation is spread within a large volume. Balch et al. investigated syntactic foams with aluminum matrix [9]. They found that in ductile matrix the damage is spread over a very large bulged region and the deformation is characterized by the plastic deformation of the cell walls, coupled with the fracture of ceramic spheres. Both, the ASK and DIC analyses suggest that the failure mechanisms revealed in Al-based MMS foam in this work agree with previous observations.

At 2% macroscopic strain, AlSi12 foam exhibits roughly the same very low level of deformation localization as in the case of Al foam. The first significant deformation band appears only at 4% strain, at an angle of ~45° [28]. At this strain level, the sample starts to crush within this highly-localized deformation band owing to the brittleness of the alloyed Al-Si matrix, thus confirming the results discussed above.

At last, the results of this study show that the ASK analysis of AE data, combined with SEM, video, and DIC analyses, is particularly useful in monitoring of the dynamics of deformation behavior (i.e., the identification of dominant deformation and fracture mechanisms during loading). This, in turn, might allow designing MMC foams better suitable for given applications.

## 4. Conclusions

The differences in deformation and failure mechanisms were investigated in two types of metal matrix syntactic foams consisting of (i) commercially pure Al matrix or (ii) Al-12 wt % Si (AlSi12) eutectic alloy matrix, both reinforced with hollow ceramic spheres. The ASK analysis of AE data (supplemented by imaging techniques) was used in this study and proved to be a powerful method to characterize active deformation mechanisms on a microscopic level. The deformation curves and the ASK analysis of AE data revealed that both foams deform, after a short elastic region, by plastic deformation of the matrix material. In addition, the fracture of brittle phases in the matrix was observed in AlSi12 foam at low stresses. It was shown that the strain localization in the latter stages of deformation is present in both foam types: broad deformation bands caused by a collective collapse of cell walls were observed in pure Al foam, whereas characteristic brittle fracture of the AlSi12 foam matrix resulted in distinctive, highly localized bands. The hollow spheres started to fracture roughly at the same stress level (60 MPa) in both foam types.

## Supplementary Materials

The following materials are available online at www.mdpi.com/1996-1944/10/2/196/s1.
