# Supplementary material for: The Effect of Matrix Composition on the Deformation and Failure Mechanisms in Metal Matrix Syntactic Foams during Compression
Source: Materials (Basel). 2017 Feb 17;10(2):196. doi: 10.3390/ma10020196 (PMC5459142; doi:10.3390/ma10020196)
Supplement: Supplementary file 1 [file materials-10-00196-s001.pdf]

Supplementary data have been uploaded to Google Drive: http://bit.ly/2kbLTGH
